# Oral low dose of glutamine improved the spontaneous closure in patients with external duodenal fistula: a retrospective comparative study with propensity score matching

**DOI:** 10.3389/fnut.2025.1614782

**Published:** 2025-07-02

**Authors:** Ming Huang, Shikun Luo, Zheng Yao, Xin Xu, Risheng Zhao, Yunzhao Zhao, Tianchi Yu

**Affiliations:** Department of General Surgery, Jiangning Hospital, Nanjing, China

**Keywords:** glutamine, fistula, spontaneous closure, outcomes, length of stay

## Abstract

**Background:**

Spontaneous closure of external duodenal fistula (EDF) is associated with reduced gastrointestinal inflammation. Low-dose glutamine supplementation in the intestine can directly improve intestinal permeability and promote mucosal healing, potentially aiding fistula closure. This study investigates the effects of oral glutamine supplementation on EDF treatment outcomes.

**Methods:**

A retrospective cohort study was conducted from January 2019 to June 2024, including 104 adult patients with EDF. Based on the administration of low-dose (10 g/day) oral glutamine supplementation, patients were divided into a glutamine group (*n* = 54) and a non-glutamine group (*n* = 50), with 46 matched pairs after propensity score matching (PSM). The outcomes were spontaneous fistula closure, mucosal healing, and hospital stay duration.

**Results:**

Spontaneous closure occurred in 28 patients (52%) in the glutamine group, compared to 21 patients (42%) in the non-glutamine group (*p* = 0.04). Glutamine promoted fistula closure both before (HR = 1.82; 95% CI: 1.03–3.23, *p* = 0.04) and after PSM (HR = 1.94; 95% CI: 1.07–3.53, *p* = 0.03). The median hospital stay was shorter in the glutamine group, both before [65 days (IQR: 32–121 days) vs. 106 days (IQR: 56–119 days), *p* = 0.01] and after PSM [76 days (IQR: 32–122 days) vs. 110 days (IQR: 54–122 days), *p* = 0.02].

**Conclusion:**

Glutamine supplementation may enhance spontaneous EDF closure and reduce hospital stay duration.

## 1 Introduction

External duodenal fistula (EDF) is a complex and life-threatening condition that can result from various etiologies, including surgical procedures, abdominal trauma, pancreatitis, and ulcers (1). Previous studies have suggested that spontaneous closure of the fistula is associated with a reduction in gastrointestinal (GI) inflammation (2–4). Glutamine, a non-essential amino acid, plays a controversial role in the treatment of various diseases, with its effectiveness varying depending on dosage and duration (5). However, the benefits of low-dose glutamine supplementation have been observed in gastrointestinal diseases. Zhou et al. (6) demonstrated that low-dose glutamine supplementation significantly improves intestinal cell connectivity and safely reduces key endpoints related to irritable bowel syndrome (IBS). Benjamin et al. (7) also reported that low-dose glutamine enhances intestinal permeability and morphology in patients with Crohn’s disease, primarily affecting the ileum and upper gastrointestinal tract. This action is attributed to its ability to mitigate the inflammatory response. Glutamine has been shown to reduce the levels of pro-inflammatory cytokines (8, 9), while promoting the production of anti-inflammatory cytokines, thereby enhancing GI mucosal healing and facilitating the closure of GI fistulas. Additionally, when administered via the digestive tract, glutamine can act directly on the intestinal mucosa without being absorbed, providing mucosal protection (10, 11). Consequently, we hypothesize that in patients with EDF, oral administration of low-dose glutamine can directly act on the mucosa at the fistula site to reduce inflammation, thereby increasing the likelihood of spontaneous closure and promoting mucosal healing.

## 2 Materials and methods

This retrospective cohort study was conducted at our hospital, adhering to the ethical principles outlined in the Declaration of Helsinki.

### 2.1 Treatment of EDF

The treatment of EDF followed the SOWATS treatment guidelines (12), which include components such as sepsis management, optimization of nutritional status, wound care, anatomical assessment of the leakage, timing of surgery, and surgical strategy. In this study, patients were required to fast throughout the treatment process. Total parenteral nutrition (PN) was administered with a target caloric intake of 30 kcal/kg/day. Gastroscopy was performed as early as possible on the basis of infection control to observe the intestinal wall defect under endoscopy and place the nasointestinal tube for enteral nutrition (EN). For high-output fistulas, duodenal fluid reinfusion was performed via the nasointestinal tube. When EN could provide more than 60% of the target caloric intake, PN was gradually weaned off. Antibiotics were discontinued when patients were free from systemic inflammatory response syndrome (SIRS) and exhibited normal white blood cell (WBC) and procalcitonin (PCT) levels. A definitive surgery (DS) was considered when the following criteria were met: (1) C-reactive protein (CRP), WBC, and PCT remained normal for over one month; (2) body mass index (BMI) ≥ 18.0 with normal physical strength, hemoglobin ≥ 100 g/L, and albumin ≥ 30 g/L; (3) an interval exceeding three months post-admission. During the preoperative three-month period, patients who demonstrated spontaneous closure before DS were allowed to self-direct their eating and were subsequently discharged.

### 2.2 Glutamine usage

Glutamine powders were provided before March 2022 in our hospital. After infection resolution, patients received glutamine powders (2.5 g each time, dissolved in 5 mL of saline solution, 4 times a day) throughout the treatment period until DS for the fistula. Subsequently, glutamine powders was stopped supply at our center. The usage was terminated.

### 2.3 Mucosal morphology and EDF

Iodinated contrast agents were used to diagnose duodenal fistulas. A pressure of 60 PSI was applied to the drainage tube, and the contrast agent was injected through it to confirm the presence of the fistula and identify its location. While abnormalities in mucosal morphology are common in intestinal fistulas, isolated mucosal abnormalities are not diagnostic of fistulas. Furthermore, in our patients, pressurized contrast imaging revealed evidence of the fistula, whereas endoscopy showed no mucosal changes. This discrepancy may be due to the fistula being small and located within a mucosal fold, making it undetectable by endoscopy.

### 2.4 Patients and outcomes

Adult patients with EDF after infection control (glutamine is only used after infection control) from January 2019 to June 2024 were included in the study. Patients were followed up from admission to discharge or DS. Exclusion criteria included: (1) patients unable to use glutamine throughout treatment due to temporary medication discontinuation; (2) patients receiving digestive tract diversion preventing direct drug action on the duodenum (stump fistula). Based on glutamine usage, patients were divided into the glutamine group and non - glutamine group. The primary outcome was the spontaneous closure during hospitalization, confirmed by upper gastrointestinal imaging showing the absence of intestinal fluid in the drainage tube. Secondary outcomes included mucosal healing and length of hospital stay. After confirming spontaneous closure through gastrointestinal imaging, complete normality of the duodenal mucosa (without defects, erosion or ulcer) was observed under gastroscopy, which is called mucosal healing.

### 2.5 Data collection and statistical analysis

During the uncontrolled infection period post - admission, patients underwent laboratory tests at least every 48 to 72 h, and every 4–7 days after infection control. CT scans were performed at intervals of no more than seven days during sepsis. The visceral fat area (VFA), total abdominal muscle area index (TAMAI), and subcutaneous fat area (SFA) values were derived from the most recent CT scan conducted after the cessation of antibiotics. The VFA/SFA and VFA/TAMAI ratios were calculated using abdominal CT images processed with ImageJ software (NIH, Bethesda, MD, USA). For each patient, two consecutive axial CT images at the level of the inferior endplate of the L3 lumbar vertebra were processed and averaged. Endoscopic mucosal morphology was evaluated at least twice during the treatment: at admission and when DS was planned or spontaneous closure was suspected. Due to the lack of standardized endoscopic evaluation criteria for enterocutaneous fistulas, we document endoscopic mucosal erosion and identifiable intestinal wall defect as clinical characteristics. Blood glucose levels were controlled below 10 mmol/L during the whole treatment (10). The Statistical Package for the Social Sciences version 26.0 for Windows (IBM, Analytics, Armonk, NY) and R.4.4.1 were used to perform statistical analyses. The Mann–Whitney U and Kruskal–Wallis tests were employed for continuous variables. Fisher’s exact test was used for categorical variables. Fisher’s exact test was carried out to compare categorical variables. Followed by a log-rank test and multivariate Cox regression analysis, Kaplan–Meier estimates were referenced to compare the effects produced by different methods. A 1: 1 Propensity score-matching (PSM) was used to reduce the impact of treatment-related bias on the practice of estimating the treatment effects with observational data. The patients in the PSM groups were matched on the basis of calculated propensity scores by a regression model with demographic data, fistula characteristics, patients condition on the day of admission, laboratory test on the day of getting rid of antibiotics, and comorbidity as covariates. The match tolerance was set to 0.2. A smaller *p* value than 0.05 was treated as statistically significant. Diagnosis of spontaneous closure can be confirmed by upper gastrointestinal imaging after the absence of intestinal fluid in the drainage tube.

## 3 Results

### 3.1 Population and characteristics

A total of 140 patients with EDF were treated at our center. After excluding 36 patients [8 who were unable to use glutamine throughout the treatment and 28 who underwent digestive tract diversion (Billroth II or Roux-en-Y)], 104 patients were enrolled in the study, with no reported deaths. The median age of these patients was 44 years (IQR: 38–48 years), and the median BMI was 20.3 kg/m^2^ (IQR: 19.3–22.1 kg/m^2^). Males comprised 59.6% (*n* = 62) of the study population. The etiologies of EDF included perforated ulcers (*n* = 7), pancreatitis (*n* = 67), trauma (*n* = 25), and endoscopic retrograde cholangiopancreatography (ERCP) (*n* = 5). The fistulas were located in the bulb (*n* = 24), descending (*n* = 68), and horizontal (*n* = 21) portions of the duodenum. Mucosal erosion or ulcers were detected in all 104 patients, and 46 patients also had an intestinal wall defect (Table 1).

**Table 1:** Baseline characteristics of enrolled patients.

Characteristics	Total (*n* = 104)	Before PSM			After PSM		
		Glutamine group (*n* = 54)	Non - glutamine group (*n* = 50)	*P*	PSM glutamine group (*n* = 46)	PSM Non - glutamine group (*n* = 46)	*P*
Demography							
Male, No. (%)	62 (59.6)	31 (57.4)	31 (62)	0.63	25 (54.3)	29 (63.0)	0.39
Age, year (median, IQR)	44 (38–48)	44 (38–48)	44 (39–48)	0.68	44 (38–48)	44 (38–48)	0.75
BMI, kg/m^2^ (median, IQR)	20.3 (19.3–22.1)	20.1 (19.2–21.6)	21.1 (19.4–22.6)	0.39	20.2 (19.5–21.9)	20.9 (19.4–22.6)	0.49
Characteristics of fistula							
Interval from fistula occurrence to admission, days (median, IQR)	20 (16–25)	20 (15–24)	21 (17–26)	0.38	20 (15–25)	20 (17–26)	0.89
Interval from admission to getting rid of antibiotics, days (median, IQR)	27 (20–32)	28 (20–33)	27 (20–29)	0.12	28 (21–32)	27 (21–29)	0.26
Intervention measures for infection control, No. (%)				0.44			0.89
Antibiotic	6 (5.8)	2 (3.7)	4 (8)		2 (43.5)	2 (43.5)	
Puncture drainage	89 (85.6)	46 (85.2)	43 (86)		42 (91.3)	41 (89.1)	
Required laparotomy	9 (8.7)	6 (11.1)	3 (6)		2 (43.5)	3 (6.5)	
High output, No. (%)	81 (77.9)	44 (81.5)	37 (74)	0.38	38 (82.6)	35 (76.1)	0.44
Etiology, No. (%)				0.68			0.83
Perforated ulcer	7 (6.7)	5 (9.3)	2 (4)		4 (86.9)	2 (43.5)	
Trauma	67 (64.4)	35 (64.8)	32 (64)		29 (63.1)	30 (65.2)	
Pancreatitis	25 (24.1)	12 (22.2)	13 (26)		11 (23.9)	11 (23.9)	
ERCP	5 (4.8)	2 (3.7)	3 (6)		2 (43.5)	3 (6.5)	
Location, No. (%)				0.75			0.56
Bulb	24 (23.1)	13 (24.1)	11 (22)		11 (23.9)	11 (23.9)	
Descender	68 (65.4)	36 (66.7)	32 (64)		32 (69.6)	29 (63.1)	
Horizontal portion	12 (11.5)	5 (9.3)	7 (14)		3 (6.5)	6 (13.0)	
Endoscopic mucosal morphology				0.96			0.83
Only mucosal erosion or ulcer	58 (55.8)	30 (55.6)	28 (56)		27 (58.7)	26 (56.5)	
Mucosal erosion or ulcer+ Detected Intestinal wall defect	46 (44.2)	24 (44.4)	22 (44)		19 (41.3)	20 (43.5)	
Patients condition on the day of admission							
Hemoglobin, g/L (median, IQR)	79 (69–88)	80 (70–88)	78 (69–88)	0.47	80 (70–88)	79 (71–88)	0.63
White blood cell, 10^9^/L (median, IQR)	1.9 (1.5–2.3)	2 (1.6–2.3)	1.8 (1.5–2.2)	0.26	1.9 (1.5–2.3)	1.9 (1.5–2.3)	0.52
Albumin, g/L (median, IQR)	30 (26–32)	30 (27–33)	29 (25–32)	0.43	30 (27–33)	29 (25–32)	0.35
C-reactive protein, mg/L (median, IQR)	79 (57–98)	78 (58–97)	79 (57–100)	0.97	78 (58–97)	79 (60–100)	0.98
Bilirubin, μmoI/L (median, IQR)	31 (19–43)	32 (23–47)	30 (19–40)	0.21	33 (23–46)	30 (19–42)	0.28
Creatinine, μmoI/L (median, IQR)	46 (31–70)	47 (29–71)	46 (32–69)	0.94	47 (32–71)	44 (33–69)	0.83
Requireing vasoactive drugs, No. (%)	39 (37.5)	19 (35.2)	20 (40)	0.61	16 (34.8)	19 (41.3)	0.52
SOFA (median, IQR)	7 (6–8)	7 (6–8)	7 (6–8)	0.58	7 (6–8)	7 (6–8)	0.72
Laboratory test on the day of getting rid of antibiotics							
Hemoglobin, g/L (median, IQR)	85 (76–100)	88 (78–100)	82 (74–102)	0.38	87 (78–98)	83 (76–104)	0.88
White blood cell, 10^9^/L (median, IQR)	0.7 (0.6–0.9)	0.7 (0.6–0.9)	0.7 (0.5–0.9)	0.85	0.8 (0.6–0.9)	0.7 (0.5–0.9)	0.92
Albumin, g/L (median, IQR)	35 (32–37)	35 (33–37)	36 (32–37)	0.91	35 (33–38)	36 (32–38)	0.88
C-reactive protein, mg/L (median, IQR)	28 (21–38)	28 (21–37)	28 (21–38)	0.94	28 (22–37)	29 (19–38)	0.78
Bilirubin, μmoI/L (median, IQR)	29 (15–37)	28 (15–37)	31 (18–38)	0.48	29 (14–38)	31 (17–39)	0.72
Creatinine, μmoI/L (median, IQR)	51 (38–63)	53 (43–63)	47 (36–62)	0.14	53 (46–63)	48 (37–62)	0.61
Body composition when released from antibiotics							
VFA/TAMAI	2.41 (1.86–3.28)	2.39 (1.85–3.24)	2.44 (1.86–3.31)	0.54	2.39 (1.85–3.24)	2.42 (1.86–3.29)	0.74
VFA/SFA	1.03 (0.75–1.37)	1.03 (0.75–1.38)	1.02 (0.74–1.36)	0.86	1.03 (0.76–1.40)	1.02 (0.74–1.36)	0.36
Comorbidity, No. (%)							
Hypertension	3 (2.9)	2 (3.7)	1 (2)	0.61	1 (2.2)	1 (2.2)	1.00
Diabetes	4 (3.8)	3 (5.6)	1 (2)	0.62	2 (4.3)	1 (2.2)	1.00

There were 54 patients in the glutamine group and 50 patients in the non-glutamine group. The clinical characteristics of the two groups were comparable (Table 1). After propensity score matching (PSM), 46 patients from each group were further analyzed, with no significant differences in characteristics observed (Table 1).

### 3.2 Primary outcome

**Figure 1:**
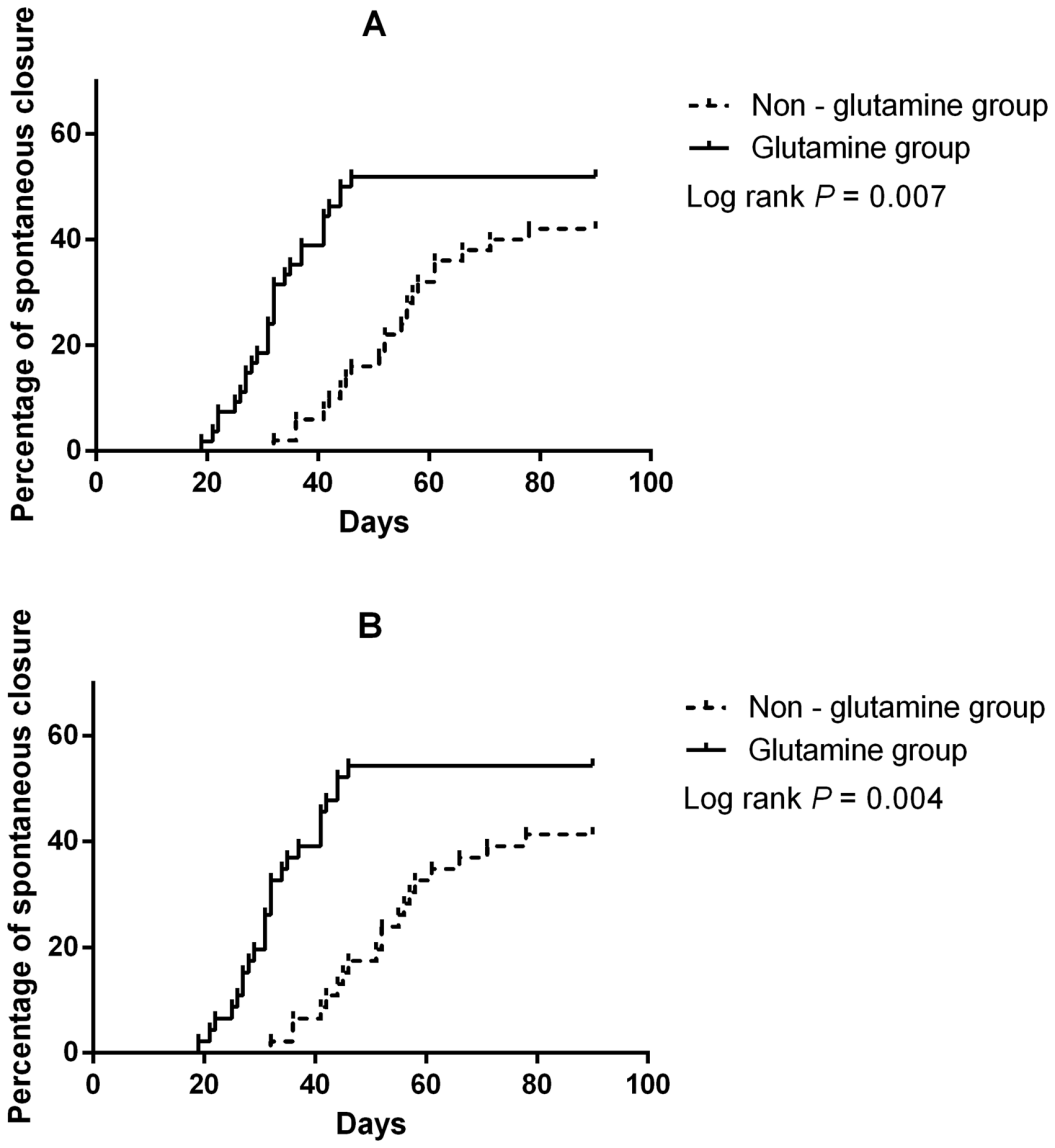
Risk factors for spontaneous closure before **(A)** and after **(B)** PSM.

**Figure 2:**
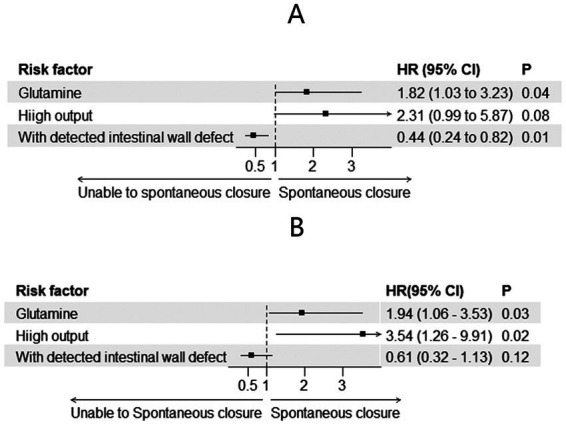
Proportion of patients with spontaneous closure before **(A)** and after **(B)** PSM.

A total of 49 patients achieved spontaneous closure (28 in the glutamine group and 21 in the non-glutamine group). The unadjusted Cox regression results are shown in Table 2. After adjusting for glutamine usage, high output, and detected endoscopic intestinal wall defects, glutamine was found to be a promoter of spontaneous closure (HR = 1.82; 95% CI: 1.03–3.23, *p* = 0.04, Figures 1A, 2A). Conversely, detected intestinal wall defects hindered spontaneous closure (HR = 0.44; 95% CI: 0.24–0.82, *p* = 0.01, Figure 2A).

**Table 2:** Unadjusted cox-regression for spontaneous closure.

Characteristics	Before PSM			After PSM		
	HR	95% CI	*P*	HR	95% CI	*P*
Glutamine	1.71	1.00–2,99	0.04	1.88	1.04–3.43	0.03
Demography						
Male	1.27	0.71–2.27	0.42	1.02	0.56–1.87	0.94
Age	0.98	0.94–1.03	0.48	0.99	0.94–1.04	0.71
BMI	1.01	0.84–1.18	0.97	1.03	0.87–1.22	0.75
Characteristics of fistula						
Interval from fistula occurrence to admission	1.00	0.96–1.05	0.92	1.01	0.97–1.06	0.60
Interval from admission to getting rid of antibiotics	0.96	0.94–1.01	0.14	0.98	0.95–1.02	0.39
Intervention measures for infection contro						
Antibiotic	Ref			Ref		
Puncture drainage	0.57	0.21–1.59	0.28	0.74	0.23–2.41	0.62
Required laparotomy	0.22	0.04–1.21	0.10	0.56	0.11–2.76	0.47
High output	2.51	1.07–5.89	0.04	3.45	1.23–9.65	0.02
Etiology						
Perforated ulcer	Ref			Ref		
Trauma	0.48	0.19–1.24	0.13	0.41	0.12–1.22	0.18
Pancreatitis	0.38	0.13–1.12	0.11	0.36	0.11–1.09	0.12
ERCP	0.39	0.08–2.07	0.27	0.32	0.07–1.99	0.24
Location						
Bulb	Ref			Ref		
Descender	0.92	0.51–1.99	0.98	0.91	0.46–1.82	0.79
Horizontal portion	0.75	0.26–2.17	0.60	0.53	0.14–1.88	0.33
Endoscopic mucosal morphology						
Only Mucosal erosion or ulcer	Ref			Ref		
Detected Intestinal wall defect	0.42	0.25–0.84	0.01	0.51	0.27–0.97	0.40
Patients condition on the day of admission						
Hemoglobin	1.00	0.98–1.03	0.82	1.01	0.98–1.03	0.62
White blood cell	1.21	0.63–2.35	0.57	1.01	0.51–2.03	0.97
Albumin	0.99	0.92–1.07	0.83	0.96	0.89–1.04	0.29
C-reactive protein	1.00	0.99–1.01	0.77	1.00	0.99–1.01	0.95
Bilirubin	0.98	0.96–1.01	0.15	0.99	0.97–1.01	0.31
Creatinine	0.99	0.98–1.00	0.27	0.99	0.08–1.00	0.65
Requireing vasoactive drugs	0.68	0.38–1.25	0.21	0.84	0.46–1.56	0.58
SOFA	0.86	0.66–1.13	0.27	0.88	0.65–1.18	0.38
Laboratory test on the day of getting rid of antibiotics						
Hemoglobin	0.99	0.97–1.02	0.49	0.99	0.97–1.02	0.96
White blood cell	0.75	0.19–2.88	0.67	0.87	0.21–3.58	0.84
Albumin	0.98	0.89–1.08	0.74	0.98	0.88–1.07	0.64
C-reactive protein	0.99	0.97–1.02	0.61	0.99	0.96–1.02	0.45
Bilirubin	0.99	0.97–1.01	0.43	0.99	0.97–1.02	0.53
Creatinine	1.00	0.97–1.03	0.91	1.00	0.98–1.03	0.71
Body composition when released from antibiotics						
VFA/TAMAI	0.77	0.58–1.02	0.06	0.74	0.54–1.02	0.06
VFA/SFA	0.79	0.34–1.05	0.15	0.88	0.53–1.48	0.64
Comorbidity						
Hypertension	2.29	0.20–26.16	0.50	2.32	0.56–9.71	0.25
Diabetes	0.95	0.36–2.82	0.69	0.92	0.22–2.71	0.67

After PSM, spontaneous closure occurred in 44 patients (25 in the glutamine group and 19 in the non-glutamine group). The unadjusted Cox regression results are also presented in Table 2. After adjusting for confounders, glutamine was again found to promote closure (HR = 1.94; 95% CI: 1.07–3.53, *p* = 0.03, Figures 1B, 2B).

### 3.3 Endoscopic mucosal morphology

**Figure 3:**
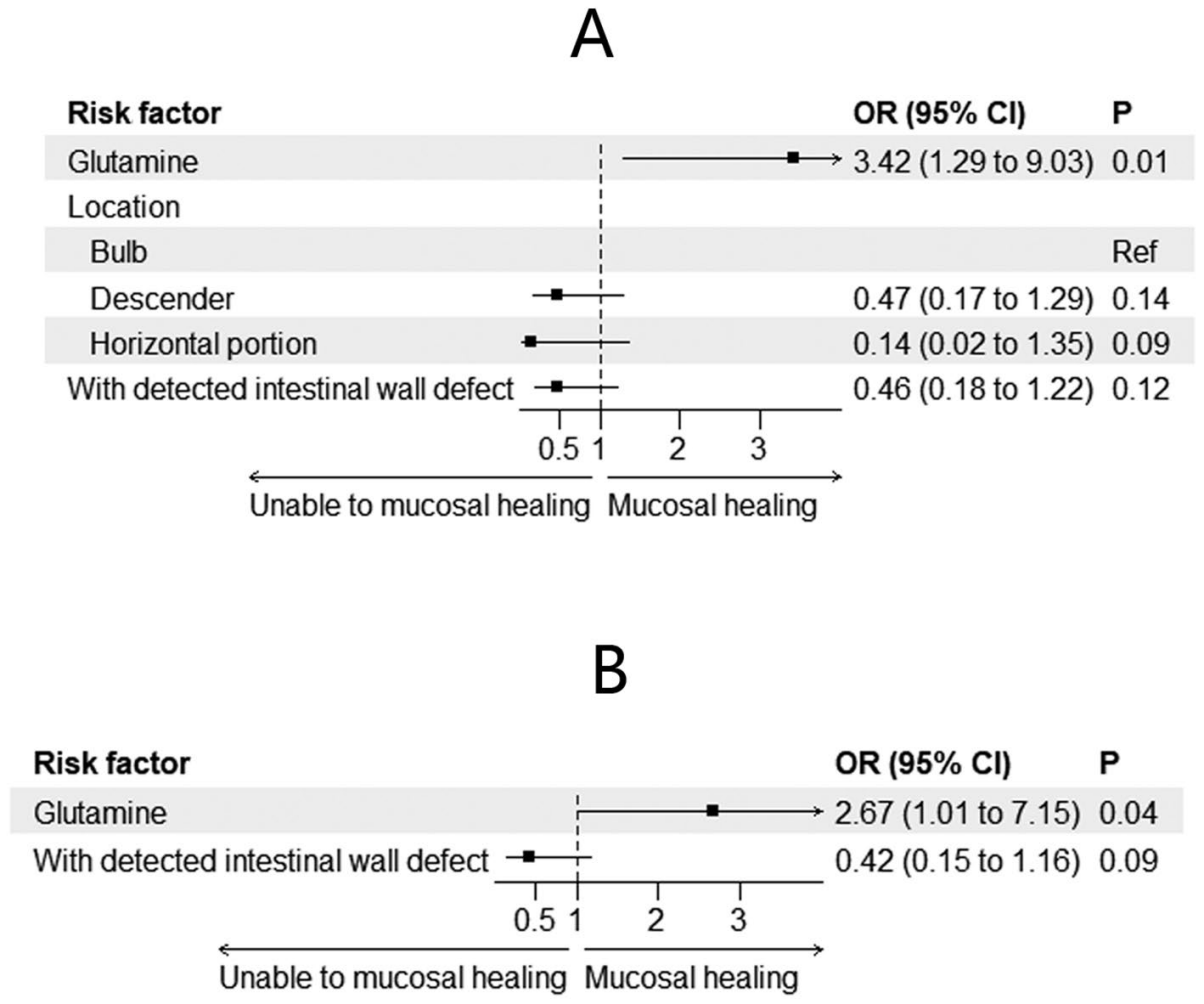
Mucosal healing before **(A)** and after **(B)** PSM.

Changes in mucosal characteristics are summarized in Table 3. Among the patients with spontaneous closure, mucosal healing was observed in 29 (59.2%) patients. The incidence of mucosal healing was higher in the glutamine group [38.2% (*n* = 21) vs. 16% (*n* = 8), *p* = 0.009]. Adjusted regression analysis revealed that glutamine promoted mucosal healing during preoperative treatment for EDF [odds ratio (OR) = 3.42; 95% CI: 1.29–9.03; *p* = 0.01, Figure 3A]. After PSM, mucosal healing was detected in 25 patients (17 in the glutamine group and 8 in the non-glutamine group, *p* = 0.04). The adjusted regression analysis again showed that glutamine promoted mucosal healing (OR = 2.67; 95% CI: 1.01–7.15; *p* = 0.04, Figure 3B).

**Table 3:** Changes in mucosal characteristics.

Mucosal characteristics on admission	Mucosal characteristics when spontaneous closure		Mucosal characteristics when DS planed in patients without spontaneous closure	
	Normal*	Erosion or ulcer	Erosion or ulcer	Intestinal wall defect
Only mucosal erosion or ulcer				
Glutamine group	18	5	7	0
Non-glutamine group	6	12	10	0
Mucosal erosion or ulcer+ Detected Intestinal wall defect				
Glutamine group	3	2	11	8
Non-glutamine group	2	1	5	14

*Patients with spontaneous closure had normal mucosa in the last gastroscope was regarded as mucosal healing.

### 3.4 Length of stay

The length of stay in the glutamine group was 65 days (IQR: 32–121 days), shorter than in the non-glutamine group, which had a median stay of 106 days (IQR: 56–119 days, *p* = 0.01). However, in patients without spontaneous closure, the length of stay was comparable between the two groups [117 days (IQR: 110–123 days) in the glutamine group vs. 119 days (IQR: 110–129 days) in the non-glutamine group, *p* = 0.41]. After PSM, the length of stay in the glutamine group was 76 days (IQR: 32–122 days), compared to 110 days (IQR: 54–122 days) in the non-glutamine group (*p* = 0.02). In patients without spontaneous closure, the length of stay was comparable [118 days (IQR: 111–124 days) in the glutamine group vs. 121 days (IQR: 110–133 days) in the non-glutamine group, *p* = 0.53].

## 4 Discussion

This study investigated the effects of low-dose oral glutamine in the treatment of EDF by analyzing a cohort of 104 patients. The results demonstrated that glutamine use was associated with a higher rate of spontaneous fistula closure, improved mucosal healing, and shorter hospital stays. These outcomes were confirmed after adjusting for confounding variables through propensity score matching (PSM). EDF represents a challenging clinical condition, with high morbidity and mortality rates, particularly when complicated by infections and sepsis (1). Spontaneous closure of fistulas is the preferred outcome, as surgical intervention carries significant risks (2, 3, 13). The literature provides limited data on the overall closure rate of EDF, with most studies focusing on duodenal stump fistulas following tumor surgery, where closure rates range from 60 to 100% (14). However, we excluded these cases from our study, as oral glutamine cannot directly reach the fistula site in stump fistulas. In our cohort, the incidence of closure was lower than that of stump fistulas, primarily due to our patient selection criteria. Theoretically, stump fistulas should exhibit lower flow rates and reduced intraluminal pressure, both of which are favorable factors for spontaneous closure. As a result, the overall lower closure rate observed in our study is consistent with these considerations. Previous studies have primarily focused on nutritional support and infection control as key factors for successful treatment. Enteral nutrition (EN) has been emphasized due to its ability to maintain gut integrity and reduce complications (13). However, less attention has been given to specific supplements, such as glutamine, in promoting mucosal healing and fistula closure. Glutamine, an amino acid with multiple roles in cellular metabolism, immune function, and intestinal barrier maintenance, has shown potential benefits in various gastrointestinal conditions, particularly in reducing inflammation and enhancing tissue repair (8, 9). The effects of glutamine vary across different diseases. A randomized controlled trial (RCT) conducted by Shariatpanahi et al. (15) found that early enteral glutamine supplementation decreased intestinal permeability in critically ill patients. Another trial demonstrated that glutamine improved outcomes in burn patients (16). A systematic review (17) indicated that glutamine supplementation, when combined with nutritional support, is associated with a significant reduction in hospital mortality and length of stay, while another review (18) suggested that glutamine supplementation did not confer significant clinical benefits in critically ill patients. Studies focused on gastrointestinal diseases have shown significant improvements in intestinal permeability and morphology with glutamine use (5). However, despite these benefits, glutamine did not improve clinical outcomes in Crohn’s disease (19). Conversely, in patients with ulcerative colitis, glutamine supplementation was associated with an improvement in overall quality of life and risk of colon cancer (19, 20). According to Holecek M (21), long-term and extensive glutamine supplementation may have side effects, while low doses appear to have no serious toxicity in healthy adults (22). Zhou et al. (6) demonstrated that glutamine supplements significantly and safely reduced major diarrhea-predominant irritable bowel syndrome (IBS)-related endpoints. Further exploration is needed to explain these phenomena. The variable results of previous studies may be associated with differences in glutamine dosage. In the present study, the glutamine dosage was relatively low compared to other studies with adverse outcomes, which often used two to three times the amount. Zhou et al. (6) achieved positive results with a dosage of 15 g/L/day, which is considered low. We believe that, in patients with EDF, small doses of oral glutamine offer two benefits: they prevent excessive intake that could lead to toxicity, and they allow glutamine to act directly on the digestive tract. After oral administration, most unabsorbed glutamine can directly affect the duodenum, altering permeability, modifying intestinal matrix connections, and reducing local inflammatory reactions in the mucosa. These effects are beneficial for the healing of duodenal fistula mucosa, which plays a key role in fistula closure and contributes to reduced hospital stays. This study has certain limitations that should be acknowledged. First, the retrospective design may introduce selection bias, despite efforts to mitigate this through propensity score matching. Second, due to the single-center nature of this study, the findings may not be generalizable to broader patient populations or different healthcare settings. Third, while the study hypothesizes that glutamine has an anti-inflammatory effect on the duodenal mucosa, it does not directly measure specific inflammatory markers or cytokines, which could provide more direct evidence of glutamine’s mechanism of action. Then, the limited sample size may affect the statistical power for some secondary outcomes, and further studies with larger, multi-center cohorts are recommended to validate these findings. Further research is warranted to explore the long-term benefits of glutamine supplementation in larger, more diverse populations, as well as its potential role in preventing fistula recurrence and improving quality of life post-discharge. Another important limitation of this study is the lack of comprehensive assessment of multiple factors that can significantly impact the closure of duodenal fistulas. These factors include cause-related factors such as the corticosteroid use, smoking, resistant bacteria, and negative nitrogen balance. Additionally, fistula-related factors, such as the length of the fistula tract, and the presence of peripheral obstruction were also not assessed. These unaddressed variables represent important limitations in understanding the full scope of influences on duodenal fistula closure. In addition, our study lacks malnutrition assessment tools. In Petra G’s latest study (23), most assessment tools have predictive value for the prognosis of abdominal surgery, with the Malnutrition Universal Screening Tool (MUST) identified as the most valuable. For surgical patients, these tools could be incorporated in future studies to optimize research strategies. Future work should also include functional measures (e.g., handgrip strength) and mechanistic studies examining adipomyokines (e.g., myostatin, leptin) to elucidate the pathways linking VFA/TAMAI to mucosal healing.

## 5 Conclusion

Oral low dosage of glutamine may improve the spontaneous closure, reducing length of stay in patients with external duodenal fistula after infection control.

## Data Availability

The raw data supporting the conclusions of this article will be made available by the authors, without undue reservation.
